# A 62-Year-Old Man With New-Onset Hypertrophic Cardiomyopathy 25 Years After Surgical Remission of Acromegaly

**DOI:** 10.1155/crie/3522275

**Published:** 2025-07-15

**Authors:** Ilan Remba-Shapiro, Cyntholia H. Okui, Sean P. Murphy, Dean Eliott, Nandita Scott, Lisa B. Nachtigall

**Affiliations:** ^1^Neuroendocrine Unit, Department of Medicine, Massachusetts General Hospital, Harvard Medical School, Boston, Massachusetts, USA; ^2^Department of Cardiology, Massachusetts General Hospital, Harvard Medical School, Boston, Massachusetts, USA; ^3^Department of Ophthalmology, Massachusetts Eye and Ear Infirmary, Harvard Medical School, Boston, Massachusetts, USA

## Abstract

Acromegaly is a rare disease that is caused by a growth hormone (GH) secreting pituitary tumor. This is a case of a 62-year-old man who presented with hypertrophic cardiomyopathy more than 25 years after surgical remission without other known etiologies of left ventricular hypertrophy. The patient initially presented at age 28 with symptoms of acromegaly and diagnosed himself, while several physicians dismissed the diagnosis. He underwent transsphenoidal surgery associated with long-term remission. At age 53, he developed palpitations, light headedness, dizziness, and chest tightness, and an echocardiogram demonstrated left ventricular hypertrophy. At age 60, cardiac magnetic resonance imaging (MRI) suggested hypertrophic cardiomyopathy, which continues to be followed. This case raises the question of whether cardiac morphological changes occur in patients with acromegaly who have GH and insulin-like growth factor-1 (IGF-1) levels well controlled. Cardiac MRI is the most accurate imaging modality for assessment of cardiomyopathy. However, more research is needed to inform clinical guidelines on screening for cardiac functional and morphological changes in patients with acromegaly.

## 1. Introduction

Acromegaly is a rare hormonal disorder that results from a growth hormone (GH)-secreting pituitary adenoma and may be associated with cardiac dysfunction [1]. There are several comorbidities of acromegaly that are associated with increased cardiovascular risk, including diabetes, hypertension, and hyperlipidemia [2]. Patients with acromegaly also have an increased risk of valvular heart disease, arrhythmias, endothelial dysfunction, and congestive heart failure [3]. Left ventricular hypertrophy may occur in acromegaly, particularly in patients with longer duration of and/or more severe disease [4–7]. A positive correlation between structural abnormalities on cardiac magnetic resonance imaging (MRI) and GH/insulin-like growth factor-1 (IGF-1) levels has been reported [8]. However, cardiac morphologic and functional changes have been described even in patients with controlled acromegaly [9]. Here, we describe a case of a patient with acromegaly in remission for 25 years who presented with cardiac symptoms and was diagnosed with hypertrophic cardiomyopathy while GH/IGF-1 levels were normal. The patient provided written informed consent.

## 2. Case Presentation

**Figure 1:**
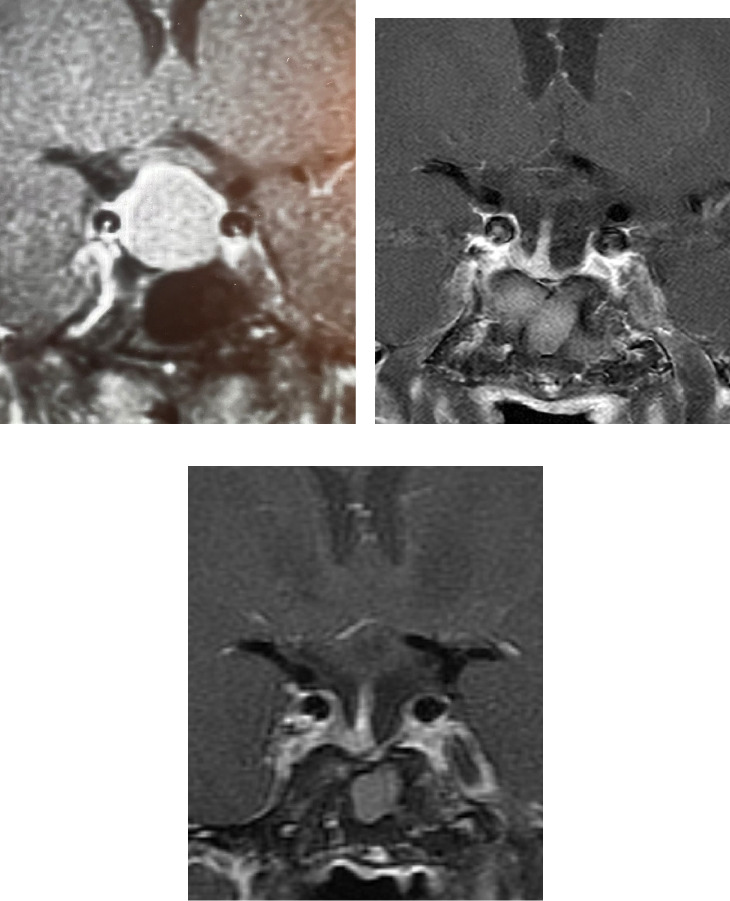
Pituitary magnetic resonance imaging. Coronal postcontrast MRIs are shown: (A) baseline prior to surgery, (B) 19 years postoperatively, and (C) 33 years postoperatively.

A 62-year-old man initially presented with bilateral carpal tunnel syndrome, acral enlargement, deepening of his voice, palpitations, and soft tissue swelling. He first noticed these changes at age 28, when he was an ophthalmology resident, and suspected the diagnosis of acromegaly. He sought an evaluation from an endocrinologist and three different orthopedists, who told him he did not have signs of acromegaly and referred him to psychiatry. He pursued his own testing which showed high GH levels (>60 ng/mL) as well as an elevated IGF-1 level (exact value unavailable). A pituitary MRI showed a 19 mm pituitary adenoma (Figure 1A), and the patient underwent transsphenoidal surgery 2 weeks later. Pathology confirmed a GH-secreting pituitary adenoma and postoperative evaluation at that time showed normalization of GH and IGF-1, intact pituitary hormonal axes and resolution of the patient's palpitations and soft tissue swelling.

At age 50, when he reestablished care, he had no obvious facial features of acromegaly, his blood pressure was 126/74 mmHg and his weight was 74.8 kg; however, he reported fatigue throughout the day and nonrestorative sleep. At this time, his IGF-1, nadir GH on oral glucose tolerance test, thyroid stimulating hormone (TSH), free thyroxine (FT4), morning cortisol, prolactin, and total testosterone were normal (Table 1). Review of the first available postoperative pituitary MRI, at age 47, showed no residual tumor (Figure 1B).

Three years after reestablishing care, the patient presented to his primary care physician and reported increased palpitations, lasting up to 10 s, associated with light headedness, dizziness, chest tightness, and was referred to cardiology. His cardiovascular exam showed clear lung auscultation, no jugular venous distention (JVD) regular heart rate and rhythm, normal S1 and S2, no S3 or S4, and no murmurs. His blood pressure and pulse were 100/60 mmHg and 68 bpm, respectively. An electrocardiogram (EKG) showed sinus rhythm and a first-degree atrioventricular block. He had normal electrolytes and normal endocrine tests, including normal suppression of GH on oral glucose tolerance test and a normal IGF-1 (Table 1). An echocardiogram (at age 53) showed mild prolapse of the posterior mitral leaflet and systolic anterior motion of the anterior leaflet with mild to moderate mitral regurgitation. The ejection fraction was 58% and the interventricular septum was 13 mm (normal <11 mm) consistent with upper septal hypertrophy. A 30-day event monitor was negative for arrhythmias or cardiovascular symptoms during this period. At age 54, a polysomnography diagnosed obstructive sleep apnea (OSA), and a multiple sleep latency test was suggestive of idiopathic hypersomnia. The patient was prescribed continuous positive airway pressure (CPAP), which was not well tolerated, and fatigue progressed.

At age 58, when he had recurrent cardiac symptoms, a long-term heart rate monitor documented short bursts of supraventricular tachycardia and ventricular bigeminy. Two years later, a cardiac MRI (at age 60) showed normal left ventricular ejection fraction, no late gadolinium enhancement, borderline asymmetric left ventricular septal hypertrophy (14–15 mm in the basal septum), and systolic anterior motion of the mitral leaflet with accompanying flow acceleration in the left ventricular outflow tract, raising the suspicion for evolving hypertrophic cardiomyopathy.

**Figure 2:**
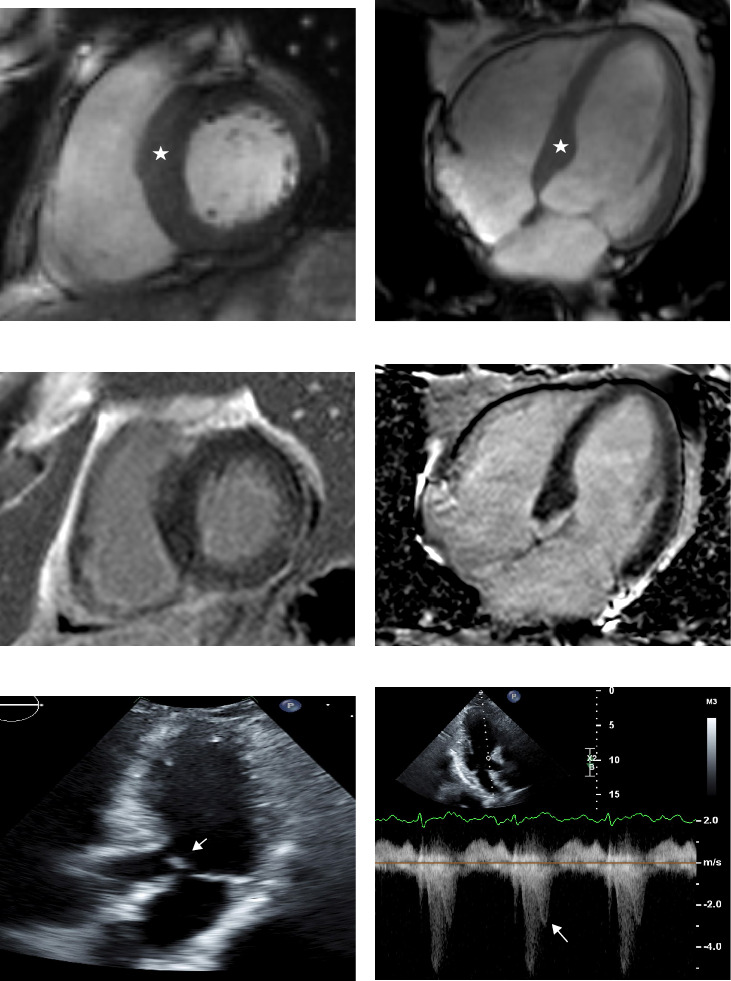
Cardiac magnetic resonance imaging and echocardiography. (A–D) Cardiac magnetic resonance imaging demonstrates asymmetric septal hypertrophy (star) (A–B) and absence of late gadolinium enhancement (C–D). (E) Transthoracic echocardiography demonstrates systolic anterior motion (SAM) of the mitral valve (arrow) with obstruction of the left ventricular outflow tract. (F) Continuous wave Doppler is consistent with dynamic left ventricular outflow tract obstruction due to SAM, with a late-peaking gradient of 37 mmHg (arrow).

At the age of 61, the patient experienced worsening of heart palpitations, bilateral pedal edema, and increased dizziness with physical activity. The diagnosis of hypertrophic obstructive cardiomyopathy was confirmed by subsequent cardiac MRI (at age 61), which documented asymmetric hypertrophy of the basal interventricular septum measuring up to 15 mm and no late gadolinium enhancement (Figure 2). One month later, he underwent an exercise stress echocardiogram. At rest, the echocardiogram showed a small left ventricle cavity, an ejection fraction of 70%, upper septal hypertrophy (15 mm), and systolic anterior motion of the mitral valve leaflets without a significant left ventricle outflow tract gradient (5 mmHg). However, with the Valsalva maneuver, both the degree of systolic anterior motion and the left ventricle outflow tract gradient (37 mmHg) increased (Figure 2). Immediate post-stress images demonstrated an increased left ventricle outflow tract gradient (50 mmHg), mild-to-moderate mitral regurgitation, and increased systolic anterior motion compared to baseline. Genetic testing for cardiomyopathy was negative, including a 30-gene panel and other causes of cardiomyopathy, such as plasma cell dyscrasias and amyloid, were excluded (normal serum protein electrophoresis and free light chain assay, respectively).

**Figure 3:**
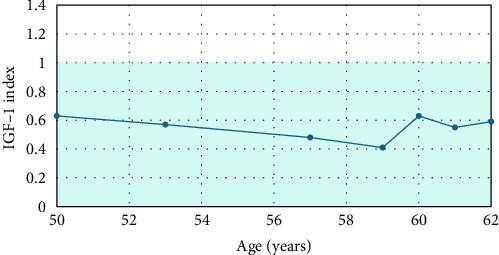
Longitudinal IGF-1 levels. The IGF-1 index is the IGF-1 level divided by IGF-1 upper limit of normal for assay (indicated by the shaded area). IGF-1 index at each year from age 50 to 62 in the patient described.

**Figure 4:**
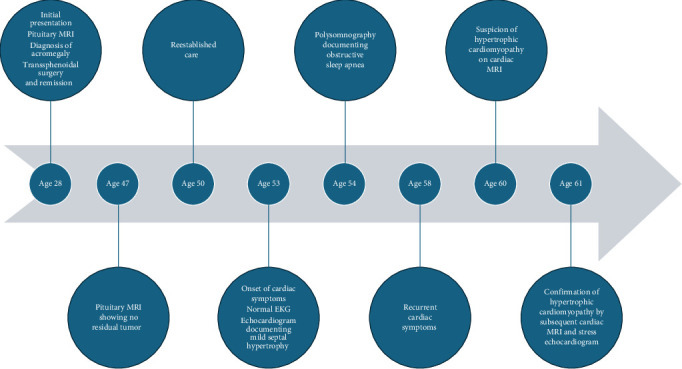
Timeline. EKG, electrocardiogram; MRI, magnetic resonance imaging.

The negative genetic testing results for cardiomyopathy do not entirely rule out the possibility of a genetic component to HCM development. However, the question of whether his cardiomyopathy could be linked to his history of acromegaly and sleep disorders remains. Interestingly, the patient denies symptoms of acromegaly, including acral enlargement, joint pain, headaches, or sweating. He has no new evidence of pituitary deficiencies and has had multiple normal IGF-1 (Figure 3) and GH levels, including oral glucose tolerance tests with appropriate suppression of GH. His blood pressure and weight have remained stable at 116/70 mmHg and 77 kg, respectively, and the cardiology team continues to observe and manage his cardiomyopathy. A timeline summarizing the clinical course is shown in Figure 4.

## 3. Discussion

In summary, this is a 62-year-old man who had a long-term remission from acromegaly and presented with cardiac septal hypertrophy more than 25 years later without other known causes of hypertrophic cardiomyopathy. Higher levels of IGF-1/GH correlate with comorbidities of acromegaly, including cardiomyopathy, making earlier diagnosis of acromegaly a priority in care [3]. It is notable in this case that four physicians dismissed the diagnosis of acromegaly, which was made only because the patient, who was in the medical field, persisted in pursuing GH and IGF-1 testing. Limited data are available on structural and functional cardiac changes in patients with well-controlled acromegaly [9]. An extensive gene panel for hypertrophic cardiomyopathy was negative and his family history was noncontributory, raising the question of the role of acromegaly and its relationship to cardiac morphologic and functional changes, even though this patient had long-term remission and no recurrent GH excess. Notably, cardiac remodeling has been reported to occur early in patients with acromegaly and to progress in relation to disease activity [5]. Previous studies have shown that patients with acromegaly have increased left ventricular mass as well as other structural and functional changes compared to controls [10, 11]. Additional studies have demonstrated that myocardial remodeling may be reversible to some extent after treatment [10, 12]. However, a more recent multicenter, case–control study revealed that patients with cured or controlled acromegaly have increased left ventricular mass, left ventricular end-diastolic and end-systolic volumes, and lower right ventricular ejection fraction when compared to matched controls with similar cardiometabolic risk [9].

It is important to consider other causes of cardiac remodeling that could be present in this case. Although the patient was diagnosed with OSA, the pattern of cardiac remodeling typically seen in this condition is more consistent with pulmonary hypertension, which causes hypertrophy of the right heart cavities, rather than the septal and left ventricular hypertrophy observed in this case [13]. Furthermore, the lack of gadolinium enhancement on cardiac MRI, absence of bi-atrial enlargement on echocardiography, and a normal free light chain assay all point away from cardiac amyloidosis [13]. Finally, while a normal serum protein electrophoresis helps rule out plasma cell dyscrasias [14], the normal genetic panel does not completely exclude a genetic cause for this patient's hypertrophic cardiomyopathy as very rare genetic variants might still exist [15].

The patient's initial cardiac MRI at age 60 showed borderline septal hypertrophy and suggested left ventricular outflow tract obstruction. The cardiac MRI allowed the diagnosis of hypertrophic cardiomyopathy by identifying morphological changes. Several studies have shown that the cardiac MRI has higher accuracy than the echocardiogram in diagnosing hypertrophic cardiomyopathy, and thus the cardiac MRI is the gold standard diagnostic testing modality for cardiac imaging [8, 12, 16]. Anatomic thoracic skeletal abnormalities such as kyphosis, which can occur in patients with acromegaly, may make assessment with echocardiogram even less reliable in this subset of patients [17].

This patient's sleep disorder may contribute to the disruption of the cardiomyocyte circadian clock, which could activate GH/IGF-1 signaling and may be linked to hypertrophic cardiomyopathy [18]. Additionally, the pulsatile nature of GH, with an increased sleep amplitude, may be relevant in identifying the relationship between sleep disorders and abnormal cardiac morphologies in patients with acromegaly [19]. The genetic disruption of the cardiomyocyte circadian clock, in conditions such as OSA and idiopathic hypersomnia, may provide evidence for the association with adverse cardiac remodeling and contractile dysfunction [18]. It is also plausible that the patient's presentation of hypertrophic cardiomyopathy is not related to acromegaly, but secondary to a genetic mutation which was not captured in the genetic panel.

## 4. Conclusion

The cause of cardiomyopathy in this case is not known. It cannot be definitively attributed to acromegaly, which remains in remission and had been for many decades. In fact, the development of hypertrophic cardiomyopathy could be coincidental and unrelated in this case. However, there is a possibility that a period of GH excess, well preceding his diagnosis of cardiomyopathy, contributed to the development of cardiac structural changes. In fact, known myocardial changes can occur, even in patients with well-controlled acromegaly [9]. Additionally, after surgical remission, preexisting cardiac morphologic, and functional cardiac abnormalities do not always normalize [3]. This case is an example of the coexistence of acromegaly and cardiomyopathy and may reflect that cardiac morphological and functional changes may occur even in well-controlled patients with acromegaly. Screening for cardiac morphologic changes in patents with acromegaly who present with cardiac symptoms may allow early detection and management of hypertrophic cardiomyopathy.

## Figures and Tables

**Table 1:** Endocrine biochemical results: longitudinal follow-up.

Laboratory test	Years after surgical remission				Normal range
	22 years	25 years	29 years	33 years	
IGF-1	227 (90–360) ng/mL *29.7 (11.8–47.1) nmol/L*	180 (50–317) ng/mL *23.5 (6.5–41.4) nmol/L*	153 (50–317) ng/mL *20 (6.5–41.4) nmol/L*	154 (41–279) ng/mL *20.1 (5.4–36.1) nmol/L*	Indicated at each timepoint

GH suppression test^a^	**—**	0.2 ng/mL *0.2 μg/L*	0.1 ng/mL *0.1 μg/L*	0.1 ng/mL *0.1 μg/L*	<0.4 ng/mL <*0.4 μg/L*

TSH	1.45 μIU/mL	1.27 μIU/mL	2.17 μIU/mL	1.39 μIU/mL	0.40–5.00 μIU/mL

Prolactin	5.3 ng/mL *5.3 μg/L*	6.1 ng/mL *6.1 μg/L*	8.5 ng/mL *8.5 μg/L*	7.0 ng/mL *7.0 μg/L*	0.0–15.2 ng/mL *0.0–15.2 μg/L*

Morning cortisol	10.5 μg/dL *290 nmol/L*	11.8 μg/dL *326 nmol/L*	14.1 μg/dL *389 nmol/L*	13.4 μg/dL *369 nmol/L*	5–25 μg/dL *138–690 nmol/L*

Total testosterone	639 (270–1070) ng/mL *2216 (936-3710) nmol/L*	550 (249–836) ng/mL *1907 (863–2899) nmol/L*	679 (249–836) ng/mL *2354 (863-2899) nmol/L*	**—**	Indicated at each timepoint

Cholesterol	182 mg/dL *4.7 nmol/L*	187 mg/dL *4.8 nmol /L*	179 mg/dL *4.6 nmol/L*	**—**	<200 mg/dL *<5.2 nmol/L*

HDL	62 mg/dL *1.6 nmol/L*	68 mg/dL *1.8 nmol/L*	52 mg/dL *1.3 nmol/L*	**—**	35–100 mg/dL *0.9–2.6 nmol/L*

LDL	107 mg/dL *2.8 nmol/L*	110 mg/dL *2.8 nmol/L*	119 mg/dL *3.1 nmol/L*	**—**	50–130 mg/dL *1.3–3.4 nmol/L*

Triglycerides	65 mg/dL *0.7 nmol/L*	45 mg/dL *0.5 nmol/L*	40 mg/dL *0.5 nmol/L*	**—**	40–150 mg/dL *0.5–1.7 nmol/L*

*Note:* Values are presented using conventional and SI units. Italics denote the SI units.

Abbreviations: GH, growth hormone; HDL, high-density lipoprotein; IGF-1, insulin-like growth factor-1; LDL, low-density lipoprotein; TSH, thyroid stimulating hormone.

^a^nadir GH reported.

## Data Availability

Data sharing is not applicable to this article as no databases were generated or analyzed during the current study.
